# Birthweight in offspring and cardiovascular mortality in their parents, aunts and uncles: a family-based cohort study of 1.35 million births

**DOI:** 10.1093/ije/dyz156

**Published:** 2019-07-20

**Authors:** Fareeha Shaikh, Marte Karoline Kjølllesdal, David Carslake, Camilla Stoltenberg, George Davey Smith, Øyvind Næss

**Affiliations:** 1 Institute of Health and Society, Faculty of Medicine, University of Oslo, Oslo, Norway; 2 MRC Integrative Epidemiology Unit at the University of Bristol, Bristol, UK; 3 Population Health Sciences, Bristol Medical School, University of Bristol, Bristol, UK; 4 Norwegian Institute of Public Health, Oslo, Norway; 5 Department of Global Public Health and Primary Care, University of Bergen, Bergen, Norway

**Keywords:** Birthweight, parents, aunts/uncles, CVD mortality

## Abstract

**Background:**

A link between suboptimal fetal growth and higher risk of cardiovascular disease (CVD) is well documented. It has been difficult to assess the contribution of environmental versus genetic factors to the association, as these factors are closely connected in nuclear families. We investigated the association between offspring birthweight and CVD mortality in parents, aunts and uncles, and examined whether these associations are explained by CVD risk factors.

**Methods:**

We linked Norwegian data from the Medical Birth Registry, the Cause of Death Registry and cardiovascular surveys. A total of 1 353 956 births (1967–2012) were linked to parents and one maternal and one paternal aunt/uncle. Offspring birthweight and CVD mortality association among all relationships was assessed by hazard ratios (HR) from Cox regressions. The influence of CVD risk factors on the associations was examined in a subgroup.

**Results:**

Offspring birthweight was inversely associated with CVD mortality among parents and aunts/uncles. HR of CVD mortality for one standard deviation (SD) increase in offspring birthweight was 0.72 (0.69–0.75) in mothers and 0.89 (0.86–0.92) in fathers. In aunts/uncles, the HRs were between 0.90 (0.86–0.95) and 0.93 (0.91–0.95). Adjustment for CVD risk factors in a subgroup attenuated all the associations.

**Conclusions:**

Birthweight was associated with increased risk of CVD in parents and in aunts/uncles. These associations were largely explained by CVD risk factors. Our findings suggest that associations between offspring birthweight and CVD in adult relatives involve both behavioural variables (especially smoking) and shared genetics relating to established CVD risk factors.

**Key Messages**

- Offspring low birthweight (LBW) was associated with increased risk of CVD mortality in parents and in aunts/uncles.
- The established CVD risk factors contributed substantially to associations among family members with a known genetic link.
- Our findings suggest that associations between offspring BW and CVD in adult relatives involve both behavioural variables (especially smoking) and shared genetics relating to established CVD risk factors.

## Introduction

A link between suboptimal fetal growth and a higher risk of cardiovascular disease (CVD) has been demonstrated within individuals in several populations.^1–3^ Some causal models have been proposed to define a mechanism underlying this association, including intrauterine programming by epigenetic mechanisms^4^ and common genetic factors influencing both fetal growth and adult diseases.^5^ Alternatively, behavioural/environmental factors may explain the low birthweight(LBW) and CVD risk association.^6^ The importance of both genetic and shared environmental factors has been emphasized in previous research.^7–9^ Some studies report stronger association in mothers than fathers, highlighting the importance of intrauterine factors.^10^^,^^11^ Moreover, a strong genetic correlation has been found in a genome-wide association study between birthweight (BW) and coronary artery disease, blood pressure and type 2 diabetes, suggesting that the association between BW and adult disease may partly be explained by shared genetic variants.^12^

Family studies have reported inverse relationships between offspring BW and CVD mortality in both parents and grandparents, which may implicate common genetic factors.^13^^,^^14^ As anticipated, maternal smoking during pregnancy was found to be a key confounding factor,^15^ suggesting genetic and non-genetic mechanisms in the intergenerational transmission of disease risk.^9^^,^^16^^,^^17^ However, it has been notoriously difficult to separate the contribution of common genetic factors from shared behavioural/socioeconomic circumstances within a nuclear family, because these potential influences are closely linked.

Investigating the offspring BW and CVD mortality association in extended family members such as aunts/uncles provides an alternative approach to studies investigating parental offspring associations. Offspring in principle share on average 50% of their genes with their parents, and they share on average 25% of their genes with their aunts and uncles. We assume that aunts/uncles in most cases belong to households different from their nieces/nephews, and therefore are less likely to share environmental factors compared with the parents and their offspring.

The objective of this study employing data from the Norwegian Medical Birth Registry and Cause of Death Registry was to investigate if the association observed between offspring BW and parental CVD mortality can also be observed for aunts/uncles, and to explore to what extent these associations are explained by known CVD risk factors such as body mass index (BMI), blood pressure, total cholesterol and smoking. We hypothesized that if shared genes explain the BW and CVD association, we would expect a stronger offspring BW and CVD mortality association in parents than in aunts/uncles, and a similar pattern of association in all four classes of aunts/uncles.

## Methods

**Figure 1.**
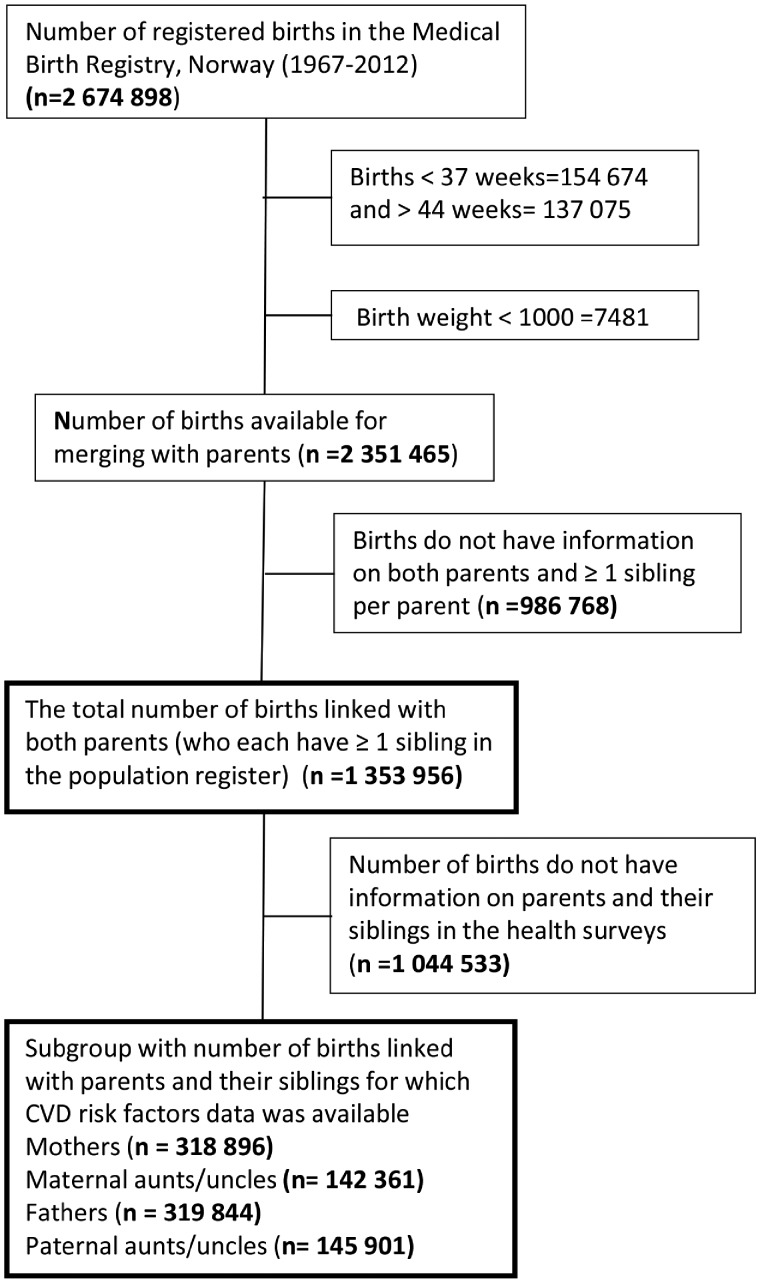
Flow chart of the study population.

A cohort was created by linking Norwegian data from cardiovascular health surveys, the Medical Birth Registry, the Cause of Death Registry, the Educational Registry and a multigenerational database containing information on familial relationships for the whole population of Norway. We included offspring (born between 1967 and 2012) with available information on their parents and at least one maternal and one paternal aunt/uncle. Aunts/uncles were defined as full siblings of a parent (sharing both mother and father). Offspring births with gestational age <37 / >44 weeks or BW <1000 g were excluded. The final dataset comprised 1 353 956 births linked to parents and one maternal and one paternal aunt/ uncle (Figure 1).

### Measures

BW (in grams) was analysed as a continuous variable and according to categories of offspring BW for gestational age: small for gestational age (SGA), <10th percentile of the BW distribution; large for gestational age (LGA), >90th percentile of the BW distribution; and appropriate for gestational age (AGA), 10th-90th percentiles of BW distribution.^18^ Additional data were included for offspring (sex, year of birth and congenital anomalies coded as ‘diseases in offspring’)^19^ and for mothers [age, parity, smoking, diseases before pregnancy (asthma, chronic hypertension, chronic renal disease, urinary tract infection, rheumatoid arthritis, heart disease, diabetes, epilepsy and thyroid diseases), and diseases during pregnancy (vaginal bleeding, glycosuria, hypertension, preeclampsia, eclampsia, gestational diabetes, anaemia, thrombosis and infection]. These maternal and offspring factors could be important confounders for the relationship between BW and CVD mortality in parents. However, to make the analysis comparable between all relationships, we adjusted model 1 for mother’s age at offspring birth in every association. Data on age at offspring's birth and the highest level of education (≤9 years, 10–12 years and ≥13 years) completed by 2011 were included both for parents and for aunts/uncles.

Three large cardiovascular health surveys—the County Study,^20^ the Age 40 Program^21^ and Cohort Norway (CONOR)^22^—were conducted in Norway during 1974–88, 1985–99 and 1994–2003, respectively. CVD risk factor data—body mass index (BMI; kg/m^2^), total cholesterol (TC; mmol/L), triglycerides (TG; mmol/L), systolic and diastolic blood pressure (SBP and DBP; mmHg), and smoking—from these health surveys were available in a subgroup (Figure 1). We used this subgroup to examine the role of traditional CVD risk factors on the association between offspring BW and CVD mortality in parents and in aunts/uncles. In the subgroup, follow-up was started from the date of CVD risk factors measurement in the population surveys.

### Outcome measure

Cause of death was acquired from the Cause of Death Registry, Norway, using the International Classification of Diseases (ICD) 8th, 9th and 10th revisions. The primary outcome was mortality from CVD (ICD 8/9: 390–459, ICD-10: 100–199). Secondary outcomes were mortality from ischaemic heart disease (IHD) and from stroke (IHD: ICD 8/9: 410–414, ICD 10: 120–125, stroke: ICD 8/9: 430–438, ICD 10: 160–169).

### Statistical analysis

Cox proportional hazard models were used to calculate the hazard ratio (HR) of deaths from CVD, IHD and stroke in parents and in aunts/uncles for a one standard deviation (SD) increase and categories of offspring BW (SGA and LGA with AGA as the reference). Parent’s, aunt’s/uncle’s age was the time axis for the Cox model. Follow-up started at the date of offspring birth and continued up to the parent’s/aunt’s/uncle’s emigration, death or end of the study (30 December 2014). The proportional hazards assumption was examined by plotting the Schoenfeld residuals and was not found to be violated by visual inspection. Total person -years included for the analysis were 30 908 031 (fathers), 31 671 408 (mothers), 29 928 884 (maternal siblings) and 30 020 262 (paternal siblings). Several offspring in our study were nested within the same parents, aunts/uncles. These offspring were clustered on their parents’ and aunts’/uncles’ identity, using the ‘vce cluster’ command in Stata. This command effectively adjusts the standard error for within-parents and within-aunts/uncles correlation. Some of the aunts/uncles appeared in the data more than once, as they could be the sibling of several mothers or fathers in the sample.

Modelling was carried out in three stages: Model 1 was adjusted for mother’s age at offspring birth (continuous). Model 2 was additionally adjusted for offspring year of birth (continuous), maternal parity (coded as 0, 1 or ≥2) and maternal diseases before and during pregnancy [coded as 0 (no) or 1 (yes) and disease in offspring at birth (coded as 0 (no) or 1 (yes)]. Model 3 was additionally adjusted for the education of parents, aunts and uncles and marital status of the parents. In the subsample for which CVD risk factor data were accessible, the association between offspring BW and mortality from CVD, IHD and stroke in parents and in aunts/uncles was first adjusted for mother’s age, which is comparable with Model 1 in the full dataset. The association was then additionally adjusted for CVD risk factors (BMI, TC, TG, SBP, DBP and smoking) and education of parents, aunts and uncles. To examine specificity of outcomes, whether the paternal association appears to reflect socioeconomic/behavioural confounding, we repeated our analysis with lung cancer mortality as outcome.

## Results

Mean follow-up time (±SD) for the parents and aunts/uncles was 47  ±  5 years. Mean age (years) at the follow-up was 54  ±  9.8 (fathers), 52  ±  9.7 (mothers), 55  ±  10.4 (maternal siblings), 56  ±  10.7 (paternal siblings). During follow-up, 0.29 % of mothers and 1.20 % of fathers died of CVD. The parents, aunts and uncles of the SGA offspring were comparatively younger and less educated than the other two groups. Maternal smoking during pregnancy was associated with lower offspring BW in the subgroup where these data were available. The maximum age of aunts and uncles at follow-up was 74 years. During follow-up, 0.55 % of maternal aunts and 1.68 % of maternal uncles died of CVD. The respective percentages for paternal aunts and uncles were 0.60 % and 1.86 % (Table 1).

**Table 1.** Characteristics of offspring, parents and aunts/uncles according to the categories of offspring birthweight

	SGA^a^	AGA^b^	LGA^c^	Overall	*P*-value
**Offspring**	**(*n* = 135 368)**	**(*n* = 1 083 163)**	**(*n* = 135 425)**	**(*n* = 1 353 956)**	
Birthweight (grams)	2.750±262	3.592±335	4.467±270	3.596±501	<0.001
Male (%)	132 981	137 383	139 635	698 589	0.482
	(51.1)	(51.2)	(51.1)	(51.1)	
Gestational age (weeks)	39.7 ±1.6	39.9 ±1.4	40.0 ±1.3	39.9 ±1.3	<0.001
Congenital diseases	3.8	3.0	3.3	3.1	<0.001
**Mothers**	**(*n* = 135 368)**	**(*n* = 1 083 163)**	**(*n* = 135 425)**	**(*n* = 1 353 956)**	
Age at offspring birth (years)	26.4±5.3	27.4±5.1	28.7±5.0	27.4±5.2	<0.001
Disease during pregnancy	11.8	6.3	6.8	6.9	<0.001
Diseases before pregnancy	6.7	6.5	8.3	6.7	<0.001
Education >13 years	29.6	38.4	42.3	36.5	<0.001
Mortality:					
CVD	0.59	0.27	0.17	0.29	<0.001
IHD	0.22	0.09	0.05	0.10	<0.001
Stroke	0.23	0.10	0.07	0.11	<0.001
Smoking during pregnancy^d^	28.0	17.1	12.7	17.3	<0.001
**Maternal aunts**	**(*n* = 62 577)**	**(*n* = 499 003)**	**(*n* = 62 538)**	**(*n* = 624 118)**	
Age at offspring birth (years)	29.5±7.5	30.4±7.4	31.7±7.4	30.5±7.4	0.002
Education >13 years	30.5	36.7	39.4	36.3	<0.001
Mortality:					
CVD	0.72	0.54	0.47	0.55	<0.001
IHD	0.33	0.21	0.16	0.22	<0.001
Stroke	0.21	0.18	0.16	0.18	<0.001
**Maternal uncles**	**(*n* = 67 201)**	**(*n* = 542 436)**	**(*n* = 67 691)**	**(*n* = 677 328)**	
Age at offspring birth (years)	29.7±7.5	30.6±7.4	31.6±7.4	30.6±7.6	<0.001
Education >13 years	24.9	29.6	30.9	29.3	<0.001
Mortality:					
CVD	2.18	1.66	1.36	1.68	<0.001
IHD	1.35	0.97	0.79	0.99	<0.001
Stroke	0.35	0.28	0.20	0.28	<0.001
**Fathers**	**(*n* = 135 368)**	**(*n* = 1 083 163)**	**(*n* = 135 425)**	**(*n* = 1 353 956)**	
Age at offspring birth (years)	29.6±5.7	30.5±5.6	31.7±5.5	30.5±5.6	<0.001
Education >13 years	24.8	31.3	33.4	30.8	<0.001
Mortality:					
CVD	1.71	1.17	0.89	1.20	<0.001
IHD	0.74	1.08	0.55	0.75	<0.001
Stroke	0.28	0.17	0.12	0.17	<0.001
**Paternal aunts**	**(*n* = 64 031)**	**(*n* = 515 151)**	**(*n* = 65 088)**	**(*n* = 644 052)**	
Age at offspring birth (years)	30.6±7.6	31.3±7.7	32.1±7.8	31.3±7.7	0.004
Education >13 years	29.4	34.2	35.4	33.70	<0.001
Mortality:					
CVD	0.92	0.57	0.45	0.60	<0.001
IHD	0.37	0.22	0.12	0.23	<0.001
Stroke	0.33	0.16	0.19	0.18	<0.001
**Paternal uncles**	**(*n* = 69 867)**	**(*n* = 556 695)**	**(*n* = 69 679)**	**(*n* = 696 241)**	
Age at offspring birth (years)	30.6±7.6	31.2±7.7	32.1±7.9	31.2±7.7	0.043
Education >13 years	24.9	28.1	28.4	27.9	<0.001
Mortality:					
CVD	2.49	1.81	1.38	1.86	<0.001
IHD	1.58	1.06	0.81	1.11	<0.001
Stroke	0.32	0.30	0.31	0.30	<0.001

a SGA (less than 10th percentile of offspring birthweight).

b AGA (10th-90th percentile of offspring birthweight).

c LGA (more than 90th percentile of offspring birthweight).

d Information on smoking during pregnancy was available in 369 844 mothers. *P*-value for continuous variables calculated by one-way ANOVA and for categorical variables by chi square test. Continuous variables are given as mean ± SD and categorical variables are given as percentages.

### Parental mortality in relation to offspring BW

An inverse association between offspring BW and age-adjusted mortality from CVD, IHD and stroke was observed among mothers and fathers, but was stronger among mothers (Table 2). For all separate causes of death, adding offspring year of birth, maternal parity, maternal ‘disease before and during pregnancy’ and ‘disease in offspring’ to the model minimally attenuated the associations in mothers and fathers (Model 2). The effect estimates for 1-SD increase in offspring BW were attenuated marginally in the parents when marital status and educational level were included in Model 3 (Table 2). The age-adjusted HR (95% CI) for CVD mortality in mothers and fathers of SGA offspring compared with AGA offspring were 2.02 (1.85–2.21) and 1.33 (1.26–1.40), respectively. In LGA offspring a reduced hazard for CVD mortality was observed among mothers and fathers [HR for mothers, 0.74 (0.63–0.86); for fathers, 0.84 (0.78–0.90)]. For IHD and stroke mortality, similar trends in SGA and LGA offspring were observed in both parents (Table 3). We also analysed data according to the sex of the offspring. No difference in association was observed in either parent (Supplementary Table 1a and b, available as Supplementary data at *IJE* online).

**Table 2.** Hazard ratio (95% CI) of deaths in parents and in aunts/uncles for 1-SD increase in offspring birthweight

		Hazard ratio (95% CI)		
	Number of deaths	Model 1	Model 2	Model 3
**Mothers^a^**				
CVD	3875	0.72 (0.69-0.75)	0.74 (0.71-0.78)	0.77 (0.74-0.80)
IHD	1351	0.69 (0.64-0.74)	0.72 (0.67-0.77)	0.75 (0.70-0.81)
Stroke	1429	0.69 (0.64-0.75)	0.71 (0.66-0.76)	0.73 (0.68-0.78)
**Maternal aunts^a^**				
CVD	3090	0.90 (0.86-0.95)	0.92 (0.88-0.97)	0.94 (0.90-0.99)
IHD	1246	0.87 (0.80-0.94)	0.88 (0.81-0.95)	0.91 (0.84-0.98)
Stroke	977	0.92 (0.85-1.00)	0.94 0.86-1.03)	0.96 (0.88-1.05)
**Maternal uncles^a^**				
CVD	10 359	0.91 (0.88-0.93)	0.92 (0.90-0.95)	0.94 (0.91-0.96)
IHD	6250	0.88 (0.85-0.91)	0.90 (0.87-0.93)	0.92 (0.89-0.95)
Stroke	1628	0.90 (0.85-0.96)	0.93 (0.81-0.99)	0.94 (0.89-1.01)
**Fathers^a^**				
CVD	16 020	0.89 (0.86-0.92)	0.90 (0.88-0.92)	0.92 (0.90-0.94)
IHD	10 090	0.88 (0.87-0.90)	0.90 (0.87-0.92)	0.92 (0.90-0.94)
Stroke	2338	0.84 (0.80-0.89)	0.86 (0.81-0.91)	0.88 (0.83-0.93)
**Paternal aunts^a^**				
CVD	3768	0.91 (0.88-0.95)	0.92 (0.89-0.96)	0.95 (0.91-0.98)
IHD	1437	0.91 (0.86-0.97)	0.92 (0.86-0.98)	0.94 (0.88-1.01)
Stroke	1225	0.89 (0.84-0.96)	0.91 (0.85-0.97)	0.92 (0.86-0.98)
**Paternal uncles^a^**				
CVD	12 697	0.93 (0.91-0.95)	0.94 (0.92-0.97)	0.95 (0.93-0.98)
IHD	7639	0.92 (0.89-0.95)	0.93 (0.91-0.96)	0.95 (0.92-0.98)
Stroke	1835	0.96 (0.90-1.02)	0.97 (0.91-1.03)	0.98 (0.92-1.05)

Model 1 was adjusted for maternal age at offspring birth. Model 2 was adjusted for Model 1 plus offspring year of birth, parity of mother, mother’s diseases before and during pregnancy, diseases in offspring. Model 3 was adjusted for Models 1 and 2 plus parental marital status and education level in parents, aunts and uncles. *P*-value for difference in effect between mother’s and father’s mortality from CVD for 1-SD increase in offspring birthweight was <0.001. *P*-values for difference in effect between maternal aunts’ and uncles’ and between paternal aunts’ and uncles’ mortality from CVD for 1-SD increase in offspring birthweight were both >0.37.

a Number of offspring linked with parents (*n* = 1 353 956), maternal aunts (*n* = 624 118), maternal uncles (*n *= 667 328), paternal aunts (*n* = 644 052), paternal uncles (*n* = 696 241).

**Table 3.** Hazard ratio (95% CI) of deaths in parents and in aunts/ uncles according to the categories of offspring birthweight

		Hazard ratio (95% CI)						
			Model 1		Model 2		Model 3	
	Number of	AGA^a^	SGA^b^	LGA^c^	SGA^b^	LGA^c^	SGA^b^	LGA^c^
	deaths							
**Mothers^d^**								
CVD	3875	1.00	2.02 (1.85-2.21)	0.74 (0.63-0.86)	1.87 (1.71-2.05)	0.76 (0.65-0.88)	1.74 (1.59-1.91)	0.80 (0.69-0.93)
IHD	1351	1.00	2.18 (1.88-2.53)	0.65 (0.49-0.86)	1.99 (1.72-2.30)	0.66 (0.50-0.88)	1.81 (1.57-2.10)	0.70 (0.52-0.92)
Stroke	1429	1.00	2.18 (1.89-2.53)	0.83 (0.65-1.05)	2.05 (1.77-2.38)	0.85 (0.67-1.08)	1.93 (1.67-2.24)	0.88 (0.69-1.12)
**Maternal aunts^d^**								
CVD	3090	1.00	1.21 (1.07-1.35)	0.96 (0.83-1.12)	1.18 (1.05-1.33)	0.97 (0.84-1.13)	1.13 (1.01-1.27)	1.00 (0.86-1.16)
IHD	1246	1.00	1.43 (1.20-1.71)	0.81 (0.62-1.05)	1.37 (1.15-1.63)	0.84 (0.65-1.10)	1.28 (1.08-1.53)	0.88 (0.68-1.15)
Stroke	977	1.00	1.14 (0.93-1.41)	0.92 (0.70-1.20)	1.09 (0.72-1.23)	0.94 (0.74-1.24)	1.03 (0.84-1.28)	0.96 (0.75-1.28)
**Maternal uncles^d^**								
CVD	10 359	1.00	1.18 (1.10-1.26)	0.92 (0.84-1.00)	1.15 (1.08-1.25)	0.94 (0.86-1.02)	1.11 (1.04-1.19)	0.95 (0.88-1.04)
IHD	6250	1.00	1.30 (1.19-1.42)	0.86 (0.77-0.96)	1.23 (1.13-1.35)	0.90 (0.80-1.01)	1.18 (1.08-1.29)	0.91 (0.82-1.02)
Stroke	1628	1.00	1.18 (1.01-1.39)	0.85 (0.64-1.01)	1.11 (0.95-1.33)	0.80 (0.63-1.00)	1.07 (0.91-1.25)	0.81 (0.64-1.02)
**Fathers^d^**								
CVD	16 020	1.00	1.33 (1.26-1.40)	0.84 (0.78-0.90)	1.25 (1.19-1.32)	0.88 (0.82-0.95)	1.19 (1.13-1.26)	0.91 (0.85-0.97)
IHD	10 090	1.00	1.33 (1.25-1.42)	0.84 (0.77-0.92)	1.26 (1.18-1.35)	0.88 (0.80-0.96)	1.20 (1.12-1.27)	0.90 (0.82-0.98)
Stroke	2338	1.00	1.53 (1.35-1.73)	0.78 (0.64-0.95)	1.45 (1.28-1.64)	0.81 (0.67-0.99)	1.38 (1.22-1.57)	0.84 (0.69-1.02)
**Paternal aunts^d^**								
CVD	3768	1.00	1.16 (1.04-1.29)	0.79 (0.69-0.91)	1.11 (0.99-1.23)	0.83 (0.72-0.95)	1.05 (0.94-1.17)	0.85 (0.74-0.97)
IHD	1437	1.00	1.20 (1.02-1.42)	0.70 (0.55-0.88)	1.18 (0.99-1.40)	0.72 (0.57-0.91)	1.11 (0.94-1.32)	0.74 (0.58-0.92)
Stroke	1225	1.00	1.16 (0.96-1.40)	0.85 (0.68-1.07)	1.14 (0.94-1.38)	0.88 (0.70-1.10)	1.10 (0.91-1.33)	0.89 (0.71-1.12)
**Paternal uncles^d^**								
CVD	12 697	1.00	1.18 (1.07-1.21)	0.87 (0.81-0.94)	1.11 (1.04-1.18)	0.91 (0.84-0.98)	1.08 (1.02-1.15)	0.92 (0.86-1.00)
IHD	7639	1.00	1.22 (1.04-1.21)	0.94 (0.84-1.01)	1.16 (1.07-1.25)	0.93 (0.85-1.03)	1.12 (1.04-1.21)	0.94 (0.86-1.04)
Stroke	1835	1.00	1.09 (0.88-1.23)	0.81 (0.66-0.99)	1.03 (0.88-1.22)	0.85 (0.70-1.06)	1.00 (0.84-1.18)	0.86 (0.70-1.06)

Model 1 was adjusted for maternal age at offspring birth. Model 2 was adjusted for Model 1 plus offspring year of birth, parity of mother, mother’s diseases before and during pregnancy, diseases in offspring. Model 3 was adjusted for Model 2 plus parental marital status and education level in parents, aunts and uncles.

a AGA (10th-90th percentile of the birthweight).

b SGA (less than 10th percentile of the birthweight).

c LGA (more than 90th percentile of the birthweight).

d Number of offspring linked with parents (*n* = 1 353 956), maternal aunts (*n *= 624 118), maternal uncles (*n* = 667 328), paternal aunts (*n* = 644 052), paternal uncles (*n* = 696 241).

### Aunts’ and uncles’ mortality in relation to niece/nephew BW

Mortality from CVD and IHD was inversely associated with offspring BW for all four classes of aunts/uncles (Table 2). For stroke mortality, there was no strong evidence that the four classes of aunts/uncles differed from each other and, individually, there was evidence weakly suggesting a negative association for all four. The strength of association was smaller in all aunts /uncles than that observed among mothers. Mortality associations in aunts/uncles were only slightly weaker than in the fathers (with largely overlapping CI). Adjustment for offspring year of birth, maternal parity, maternal diseases before and during pregnancy and disease in offspring (Model 2) minimally changed the hazard ratio for CVD and IHD mortality in all aunts/uncles. Estimates were attenuated a little in all four classes of aunts/uncles when their educational status was added as a covariate (Model 3). For CVD and IHD mortality, a higher hazard was observed in aunts/uncles of SGA offspring whereas a reduced hazard was noted in aunts/uncles of LGA offspring. For stroke mortality, results were mostly in the same direction as for CVD and IHD, but considerably weaker, with 95% CI including the null (Table 3).

**Figure 2.**
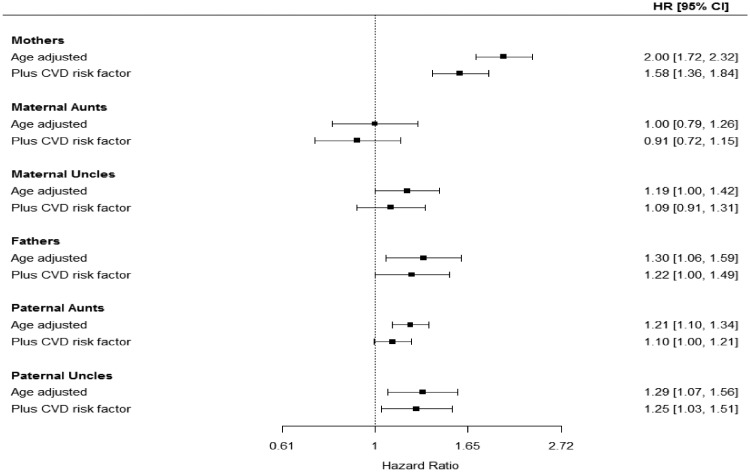
Hazard ratio (95% CI) of CVD deaths in mothers, fathers, aunts and uncles in small-for-gestational-age offspring after adjustment for CVD risk factors. Number of offspring linked with mothers (*n* = 318 896), maternal aunts (*n* = 71 727), maternal uncles (*n *= 70 634), fathers (*n *= 319 844), paternal aunts (*n* = 73 420), paternal uncles (*n* = 72 481).

In the subsample with data on CVD risk factors, an inverse association between offspring BW and CVD mortality was noted among parents and among aunts/uncles. These results were roughly comparable to the age-adjusted results in the whole dataset (Tables 2 and 3). Adjustment for CVD risk factors attenuated the associations in all relationships substantially (Table 4, Figure 2), but additional adjustment for education made a small difference to estimates. For lung cancer mortality, the patterns of results observed in parents, aunts and uncles were similar to those observed for CVD mortality (Supplementary Table 2, available as Supplementary data at *IJE* online).

**Table 4.** Hazard ratio (95% CI) of deaths in parents and in aunts/uncles according to offspring birthweight after adjusting for CVD risk factors and education. Subsample with CVD risk factors available

		Hazard ratio (95% CI)					
	Number of deaths	1-SD increase in offspring BW^a^			SGA^b^			
		Age-adjusted^c^	Plus CVD risk factors^d^	Plus education^e^	Age-adjusted^c^	Plus CVD risk factors^d^	Plus education^e^
**Mothers^f^**							
CVD	1325	0.70 (0.65-0.76)	0.79 (0.73-0.84)	0.80 (0.74-0.85)	2.00 (1.72-2.32)	1.58 (1.36-1.84)	1.55 (1.33-1.80)
IHD	480	0.71 (0.63-0.79)	0.81 (0.72-0.90)	0.82 (0.73-0.91)	1.99 (1.57-2.53)	1.50 (1.18-1.91)	1.46 (1.15-1.85)
Stroke	493	0.65 (0.57-0.73)	0.74 (0.65-0.83)	0.74 (0.66-0.84)	2.22 (1.74-2.83)	1.76 (1.38-2.24)	1.72 (1.35-2.19)
**Maternal aunts^f^**							
CVD	483	0.98 (0.91-1.14)	1.00 (0.93-1.24)	1.00 (0.91-1.10)	1.00 (0.79-1.25)	0.91 (0.72-1.14)	0.90 (0.71-1.13)
IHD	291	1.00 (0.86-1.33)	1.00 (0.91-1.35)	1.02 (0.92-1.37)	1.22 (0.70-2.12)	1.43 (0.81-1.30)	1.42 (0.81-1.47)
Stroke	162	1.00 (0.82-1.26)	1.01 (0.86-1.19)	1.02 (0.87-1.20)	0.87 (0.55-1.37)	0.77 (0.49-1.22)	0.76 (0.48-1.20)
**Maternal uncles^f^**							
CVD	1268	0.90 (0.84-0.97)	0.94 (0.87-1.01)	0.94 (0.88-1.01)	1.19 (1.01-1.43)	1.09 (0.91-1.31)	1.08 (0.90-1.30)
IHD	861	0.87 (0.80-0.96)	0.90 (0.82-0.99)	0.90 (0.82-0.99)	1.23 (1.00-1.50)	1.20 (0.93-1.49)	1.18 (0.95-1.42)
Stroke	218	0.85 (0.73-1.00)	0.89 (0.75-1.05)	0.89 (0.75-1.05)	1.14 (0.74-1.74)	1.00 (0.63-1.56)	1.00 (0.63-1.55)
**Fathers^f^**							
CVD	4700	0.91 (0.88-0.95)	0.96 (0.92-1.00)	0.96 (0.92-1.00)	1.30 (1.06-1.59)	1.22 (1.06-1.58)	1.22 (1.02-1.52)
IHD	3024	0.91 (0.87-0.96)	0.96 (0.91-1.01)	0.96 (0.92-1.01)	1.22 (1.08-1.38)	1.11 (0.98-1.26)	1.10 (0.97-1.24)
Stroke	697	0.86 (0.77-0.95)	0.90 (0.81-0.99)	0.90 (0.81-0.99)	1.36 (1.07-1.73)	1.25 (0.98-1.59)	1.25 (0.98-1.59)
**Paternal aunts^f^**							
CVD	1055	0.85 (0.78-0.92)	0.86 (0.78-0.93)	0.86 (0.79-0.93)	1.21 (1.09-1.33)	1.10 (1.00-1.22)	1.09 (0.99-1.21)
IHD	320	0.86 (0.78-0.94)	0.87 (0.79-0.96)	0.87 (0.79-0.96)	1.13 (0.88-1.45)	1.11 (0.87-1.43)	1.09 (0.85-1.40)
Stroke	167	0.84 (0.69-0.94)	0.85 (0.69-0.94)	0.85 (0.69-0.94)	1.89 (1.13-3.14)	1.91 (1.15-3.18)	1.90 (1.15-3.16)
**Paternal uncles^f^**							
CVD	1115	0.90 (0.81-1.00)	0.92 (0.83-1.03)	0.92 (0.83-1.04)	1.29 (1.07-1.56)	1.25 (1.03-1.51)	1.24 (1.02-1.50)
IHD	716	0.88 (0.81-0.96)	0.90 (0.83-0.98)	0.91 (0.83-0.98)	1.32 (1.03-1.68)	1.27 (0.99-1.62)	1.25 (0.98-1.60)
Stroke	170	0.84 (0.68-1.04)	0.85 (0.69-1.06)	0.86 (0.69-1.06)	1.40 (0.91-2.16)	1.36 (0.88-2.10)	1.35 (0.86-2.08)

a BW (birthweight).

b SGA (less than 10th percentile of offspring birthweight). Reference category is AGA (10th-90th percentile of birthweight).

c Adjusted for mother’s age.

d CVD risk factors (BMI, cholesterol, triglycerides, systolic and diastolic blood pressure and current smoking(coded as yes/no).

e Adjusted for mother’s age, CVD risk factors and education.

f Number of offspring linked with mothers (n = 318 896), maternal aunts (*n* = 71 727), maternal uncles (*n* = 70 634), fathers (*n* = 319 844), paternal aunts (*n* = 73 420), paternal uncles (*n* = 72 481).

## Discussion

We have shown an inverse association between offspring BW and mortality from CVD and IHD in parents and in their siblings (aunts/uncles). The association was stronger in mothers than in fathers or in aunts/uncles. There were no differences in the estimates among the four classes of aunts/uncles, and the associations among fathers were only slightly stronger than those in aunts/uncles. The associations were to a large extent explained by CVD risk factors.

### Comparison of results with previous studies and potential mechanisms

The relationship between lower offspring BW and increased risk of CVD among parents and aunts/uncles may support a genetic basis for the association. The relationship observed in parents is consistent with previous studies including both mothers and fathers,^23^^,^^24^ and with studies indicating a stronger association in mothers than in fathers.^14^^,^^25^ In contrast, another study reported similar father-offspring and mother-offspring associations for cardiovascular risk factors.^26^ To our knowledge, the association between niece/nephew BW and CVD mortality in aunts/uncles has not previously been explored. Therefore, direct comparison of our results with other studies is not possible. However, a number of multigenerational studies, reporting a strong association between grandchild BW and mortality in grandparents, support a genetic influence on the association between BW and CVD.^13–15^

CVD has a substantial genetic component and several genes, particularly those encoding glucokinase,^5^ clotting factors^27^ and angiotensinogen,^28^ have mutations that are associated with both restricted fetal growth and risk of CVD. A recent study also confirmed genetic influence on the association between LBW and adult hypertension.^29^ Additionally, it has been proposed that shared environmental factors, such as smoking, diet and socioeconomic position (SEP), also may contribute to the negative association between BW and CVD risk.^30^

To investigate the significance of shared familial factors, we extended our analyses and assessed the role of CVD risk factors in the relationship. The attenuation of offspring BW and CVD mortality association in parents and in aunts/uncles after adjustment for CVD risk factors suggests a contribution of familial factors shared not only in a nuclear family, but also in extended families. The impact of CVD risk factors such as blood pressure, lipids and obesity may support a role of shared genes, as these factors are genetically influenced.^31–33^ However, the contribution of health-related behaviours such as smoking^34^ may indicate the importance of shared environmental factors in the association. Smoking behaviour has been linked to genetic variants,^35^ but there is little evidence on shared genetic factors linking smoking and LBW. Furthermore, a role of education in BW and CVD mortality association was observed in all familial relationships. Studies have shown a higher obesity and diabetes risk in parents of offspring with higher BWs.^36–38^ However, we observed an increased CVD mortality among parents of SGA offspring but not with LGA offspring.^10^^,^^39^^,^^40^ These may be two different mechanisms. It might be possible that parental diabetes/obesity is more relevant to LGA offspring and CVD to SGA offspring.

Multiple potential mechanisms may explain the associations observed between offspring BW and CVD mortality in parents and aunts/uncles. Genetic confounding is one possible explanation, but for a purely genetic model we expect similar strength of associations in parents and half of this strength in aunts/uncles relationships. However, we found a stronger association in mothers than in fathers and aunts/uncles, suggesting that multiple potential mechanisms are involved in the mother-offspring association. First, intrauterine factors leading to LBW in offspring through malnutrition, poor placental growth and maternal pelvic restriction is one possibility.^41–43^ Second, a dual action of maternal genes, contributing to fetal growth both by gene inheritance and by affecting the intrauterine environment, could be another mechanism.^44^ Third, maternal health-related behaviours such as smoking may have a direct impact on offspring BW and the mother’s own risk of CVD.

We expect the genetic association for fathers to be twice that for aunts/uncles, and presumably the environmental/behavioural association would also be stronger. However, the associations in fathers were only a little stronger than those for aunts/uncles. This reflects that unobserved behavioural confounders, such as alcohol intake and physical activity, may be important in the paternal association. A similar trend of associations with lung cancer mortality also reflects the significance of behavioural confounders in the paternal association. Furthermore, the similar strength of associations with all classes of aunts/uncles is indicative of a genetic link. These associations may also be partly explained by environmental mechanisms, as parents and their siblings share similar home environments, dietary habits and health-related behaviours during early life. However, previous studies investigating offspring BW and parental sibling characteristics have suggested that maternal aunts but not uncles share important links with offspring BW. They propose that genetic effects from mothers are more important than paternal effects.^45^^,^^46^

A recent large-scale pedigree analysis suggests that assortative mating generates substantial apparent heritability with respect to mortality.^47^ Assortative mating might contribute to the mortality associations in our study. Another explanation could be the genetic nurturing phenomenon, suggesting that genetic and environmental mechanisms are interlinked and genetic effects can exert their impact through an environmentally mediated channel.^48^ The complete separation of environmental and genetic components that influence CVD mortality is difficult, and an interaction between these factors may further complicate our understanding.

### Strengths and weaknesses

Our study is based on data from the nationwide registers, providing a large sample size and comprehensive population coverage. We established a dataset of offspring, parents and their siblings (aunts/uncles), which provides an opportunity to study the association between BW and CVD mortality in family members at different degrees of relatedness. The ability to include data on CVD risk factors adds novelty to the study. We also calculated BW for gestational age, which gives a precise measure of intrauterine fetal growth. Moreover, detailed information on maternal health before and during pregnancy was also included from the registry data. Diet and physical activity, which could be important in the relationship between BW and CVD mortality, were not included in our study. Education level was included as an indicator of SEP. The data on smoking in pregnancy were collected in the Medical Birth Registry from 1998 onwards. Thus, only a few participants with short follow-up have this information, and the effect of smoking during pregnancy cannot be estimated.

## Conclusion

We show that offspring BW was associated with increased risk of CVD in parents and in aunts/uncles, and that established CVD risk factors contributed substantially to associations among family members with a known genetic link. This suggests that both behavioural factors, especially smoking, and shared genetic factors in extended family members, involving these established CVD risk factors, play roles in the associations.

## Supplementary Material

dyz156_Supplementary_Materials
