# Supplementary material for: Birthweight in offspring and cardiovascular mortality in their parents, aunts and uncles: a family-based cohort study of 1.35 million births
Source: Int J Epidemiol. 2019 Jul 20;49(1):205–15. doi: 10.1093/ije/dyz156 (PMC7124506; doi:10.1093/ije/dyz156)
Supplement: dyz156_Supplementary_Materials [file dyz156_supplementary_materials.zip › dyz156-suppl_data/Supplementary table.pdf]

Supplementary tables:

Table S1a: Age-adjusted hazard ratio (95% CI) of deaths in parents according to birth weight in female offspring (n=660 924)

	Hazard ratio (95% CI)				
	Number of deaths	AGA[a]	SGA[b]	LGA[c]	For 1- SD increase in offspring birth weight
Mothers					
CVD	1826	1.00	2.02 (1.80-2.27)	0.78 (0.61-0.99)	0.71(0.67-0.75)
IHD	626	1.00	2.01 (1.64-2.45)	0.68 (0.45-1.05)	0.70(0.64-0.77)
Stroke	665	1.00	2.04 (1.68-2.49)	0.65 (0.40-1.03)	0.68(0.62-0.75)
Fathers					
CVD	7884	1.00	1.33 (1.25-1.42)	0.92 (0.82-1.02)	0.88 (0.86-0.91)
IHD	4931	1.00	1.32 (1.31-1.73)	0.85 (0.74-0.97)	0.89 (0.86-0.93)
Stroke	1161	1.00	1.55 (1.32-1.81)	0.74 (0.54-1.01)	0.84 (0.78-0.90)

Table S1b: Age-adjusted hazard ratio (95% CI) of deaths in parents according to birth weight in male offspring (n=693 032)

	Hazard ratio (95% CI)				
	Number of deaths	AGA[a]	SGA[b]	LGA[c]	For 1- SD increase in offspring birth weight
Mothers					
CVD	2049	1.00	2.09 (1.85-2.37)	0.73 (0.61-0.87)	0.72(0.68-0.76)
IHD	725	1.00	2.49 (2.04-3.04)	0.61 (0.44-0.85)	0.67(0.61-0.73)
Stroke	764	1.00	2.23 (1.83-2.73)	0.92 (0.70-1.21)	0.70(0.64-0.77)
Fathers					
CVD ,	8163	1.00	1.33 (1.23-1.43)	0.85 (0.79-0.93)	0.88 (0.86-0.91)
IHD	5159	1.00	1.31 (1.19-1.43)	0.85 (0.77-0.95)	0.89 (0.86-0.92)
Stroke	1177	1.00	1.50 (1.24-1.82)	0.81 (0.64-1.02)	0.85 (0.79-0.91)

[a] AGA (between 10-90th percentiles of birth weight)
[b] SGA (less than 10th percentile of birth weight)
[c] LGA (more than 90th percentile of birth weight)
CVD (cardiovascular disease), IHD (ischemic heart disease)
SD (standards deviation)

Table S2: Hazard ratio (95% CI) of lung cancer mortality in parents and in aunts/ uncles according to offspring birth weight

		Hazard ratio (95% CI)			
	Number of deaths	AGA[a]	SGA[b]	LGA[c]	For 1- SD increase in offspring birth weight
Mothers[d]	3550	1.00	1.90 (1.73-2.09)	0.45 (0.38-0.58)	0.68 (0.65-0.71)
Maternal aunts[d]	2598	1.00	1.46 (1.31-1.70)	0.85 (0.72-1.30)	0.90 (0.82-0.92)
Maternal uncles[d]	3589	1.00	1.17 (1.05- 1.31)	0.81 (0.70-0.94)	0.97(0.87-0.95)
Fathers[d]	5608	1.00	1.32 (1.21-1.44)	0.88 (0.79-0.99)	0.87 (0-85-0.91)
Paternal aunts[d]	2391	1.00	1.26 (1.12-1.42)	0.90 (0.77-1.05)	0.91 (0.87-0.96)
Paternal uncles[d]	4409	1.00	1.12 (1.01-1.24)	0.95 (0.87-1.12)	0.97(0.93-1.01)

[a] AGA (between 10-90th percentile of birth weight)
[b] SGA (less than 10th percentile of birth weight)
[c] LGA (more than 90th percentile of birth weight)
[d] Number of offspring linked with parents (n=1 351 897), maternal aunts (n=615 959), maternal uncles (n=664 986), paternal aunts (n=633 390), paternal uncles (n= 681 061)
SD (standards deviation
