# Supplementary figures and images for: Birthweight in offspring and cardiovascular mortality in their parents, aunts and uncles: a family-based cohort study of 1.35 million births
Source: Int J Epidemiol. 2019 Jul 20;49(1):205–15. doi: 10.1093/ije/dyz156 (PMC7124506; doi:10.1093/ije/dyz156)

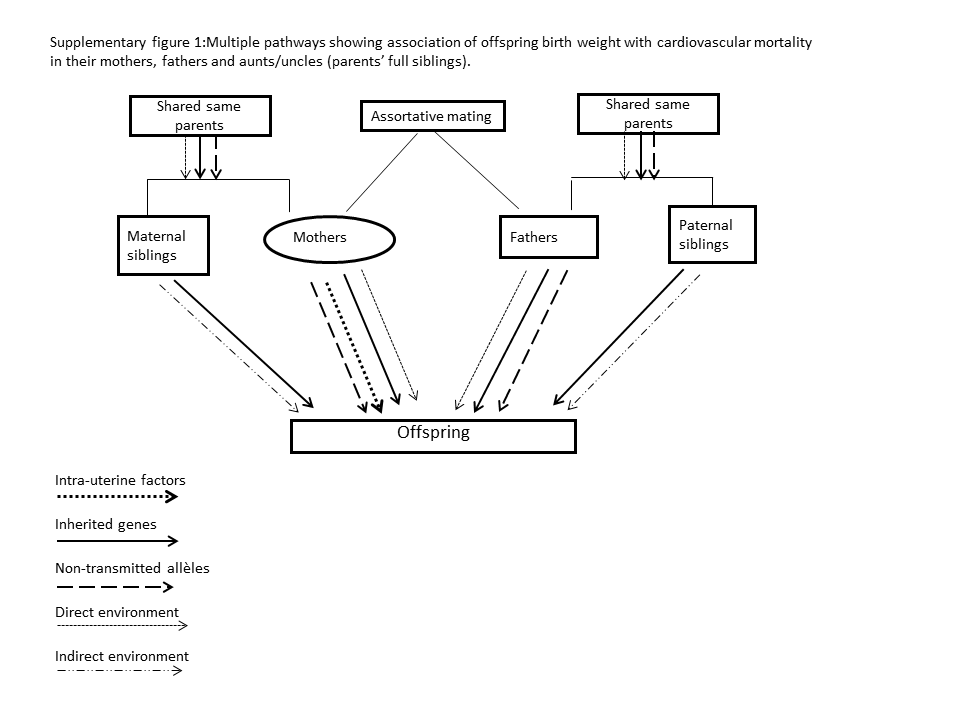

Supplement: dyz156_Supplementary_Materials [file dyz156_supplementary_materials.zip › dyz156-suppl_data/Supplementary Figure.tif]
